# Cross Comparison Between Thermal Cycling and High Temperature Stress on I/O Connection Elements

**DOI:** 10.3390/mi17010088

**Published:** 2026-01-09

**Authors:** Mamta Dhyani, Tsuriel Avraham, Joseph B. Bernstein, Emmanuel Bender

**Affiliations:** Department of Electrical and Electronic Engineering, Ariel University, Ariel 40700, Israel; avrahamt@ariel.ac.il (T.A.); josephbe@ariel.ac.il (J.B.B.); carlem@ariel.ac.il (E.B.)

**Keywords:** FPGA, BTI, I/O buffer degradation, thermal stress, PCB pads, interconnect aging

## Abstract

This work examines resistance drift in FPGA I/O paths subjected to combined electrical and thermal stress, using a Xilinx Spartan-6 device as a representative platform. A multiplexed measurement approach was employed, in which multiple I/O pins were externally shorted and sequentially activated, enabling precise tracking of voltage, current, and effective series resistance over time, under controlled bias conditions. Two accelerated stress modes were investigated: high-temperature dwell in the range of 80–120 °C and thermal cycling between 80 and 140 °C. Both stress modes exhibited similar sub-linear (power-law) time dependence on resistance change, indicating cumulative degradation behavior. However, Arrhenius analysis revealed a strong contrast in effective activation energy: approximately 0.62 eV for high-temperature dwell and approximately 1.3 eV for thermal cycling. This divergence indicates that distinct physical mechanisms dominate under each stress regime. The lower activation energy is consistent with electrically and thermally driven on-die degradation within the FPGA I/O macro, including bias-related aging of output drivers and pad-level structures. In contrast, the higher activation energy observed under thermal cycling is characteristic of diffusion- and creep-dominated thermo-mechanical damage in package-level interconnects, such as solder joints. These findings demonstrate that resistance-based monitoring of FPGA I/O paths can discriminate between device-dominated and package-dominated aging mechanisms, providing a practical foundation for reliability assessment and self-monitoring methodologies in complex electronic systems.

## 1. Introduction

Field-programmable gate arrays (FPGAs) are widely used in aerospace, automotive, industrial control, and other mission-critical applications, where long-term electrical reliability is essential. The electrical integrity of input/output (I/O) interconnection paths is particularly important, as these paths form the interface between the chip and the external environment. An I/O path typically consists of solder joints, PCB pads, package interconnects, and the on-chip transistor-level input buffer circuitry. Under thermal or electrical stress, degradation in any of these elements may appear as changes in voltage drop, current flow, or effective line resistance. Correctly identifying which element is responsible for the observed degradation is therefore critical for lifetime prediction and the development of appropriate mitigation strategies [1,2].

Package-level degradation mechanisms—such as solder joint fatigue, interconnect cracking, and thermo-mechanical wear—are commonly activated by thermal cycling and generally follow well-established models such as Coffin–Manson fatigue or logarithmic stress-accumulation laws. However, in advanced CMOS-based FPGAs, semiconductor-level transistor aging increasingly dominates system reliability, especially when the device is subjected to sustained electrical bias or elevated temperature. Transistor degradation is primarily driven by three mechanisms: bias temperature instability (BTI) [3], hot-carrier injection (HCI) [4], and time-dependent dielectric breakdown (TDDB) [5]. BTI and HCI manifest as a progressive shift in the Vth due to charge trapping or interface-state generation, whereas TDDB results from progressive oxide weakening that leads to increased gate leakage [6].

The Xilinx Spartan-6 family, fabricated in a 45 nm CMOS technology [7,8], incorporates NMOS and PMOS transistors into its I/O buffer circuitry. These devices are continuously biased during operation and are therefore susceptible to both negative BTI (NBTI) in PMOS devices and positive BTI (PBTI) in NMOS devices. BTI involves charge trapping in the gate oxide, defect activation, and interface-bond breaking, producing a voltage-dependent power-law time evolution in Vth. As Vth increases, the input buffer becomes weaker, which manifests as a measurable increase in the on-state resistance and a corresponding change in the voltage drop across the I/O signal path. Several studies have examined reliability effects in SRAM-based FPGAs, including soft errors and mitigation techniques [9]; most prior work has focused on core logic or configuration memory. In contrast, the degradation behavior of I/O buffer transistors—particularly under combined thermal and bias stress—remains significantly less explored.

Recent studies have emphasized the importance of aging effects in modern FPGA platforms, particularly as continued technology scaling places stricter constraints on long-term reliability in safety and mission-critical applications. Prior work has demonstrated that transistor-level degradation mechanisms, such as bias temperature instability (BTI), hot-carrier injection (HCI), and time-dependent dielectric degradation, can significantly influence electrical behavior, timing margins, and long-term device stability in advanced CMOS technologies [3,4,5,6]. These effects become increasingly relevant as device dimensions shrink and operating margins tighten.

However, most existing investigations have primarily focused on core logic resources, routing fabrics, or configuration memory, while the aging behavior of FPGA I/O buffer circuitry remains comparatively less explored. I/O structures operate under distinct electrical and thermal conditions, due to their thicker gate oxides, different biasing schemes, and direct exposure to external environments. As a result, their degradation behavior may differ substantially from that of internal logic elements, particularly under prolonged electrical stress and elevated temperature conditions [6].

Despite this, systematic experimental studies that isolate I/O buffer degradation and distinguish transistor-level aging from package-related effects—such as solder fatigue or thermo-mechanical strain—remain limited. The present work addresses this gap by providing a focused experimental investigation of FPGA I/O buffer aging under both constant high-temperature stress and thermal cycling. Using resistance-based monitoring combined with Weibull analysis and activation-energy extraction, this study enables clear differentiation between underlying physical degradation mechanisms.

### 1.1. CMOS and Tri-State Architecture of Spartan-6 I/O Blocks

**Figure 1:**
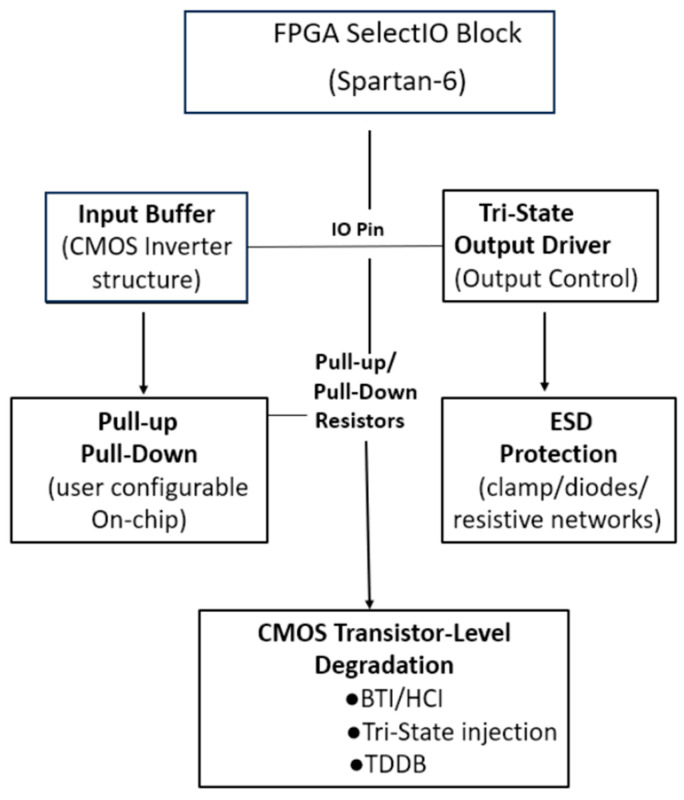
Simplified internal architecture of the Spartan-6 SelectIO pin, showing the CMOS input buffer, tri-state output driver, output-enable control, programmable pull-up/pull-down network, and ESD protection structures. These elements form the complete electrical path (R_path_) between the external pin and the internal logic and represent the MOSFET-based components that are susceptible to BTI, HCI, and TDDB degradation under thermal and electrical stress.

Modern SRAM-based FPGAs such as the Xilinx Spartan-6 (Xilinx, San Jose, CA, USA) are fabricated using a 45 nm CMOS process, in which every configurable logic block, routing switch, and user I/O pin is implemented by using complementary MOSFET structures. The SelectIO block at each FPGA pin contains a CMOS input buffer, a tri-stateable CMOS output driver, programmable slew-rate and drive-strength control, and an array of ESD protection devices (Figure 1). The tri-state output stage consists of a pull-up PMOS transistor and a pull-down NMOS transistor, together with an enable transistor pair that electrically disconnects the driver when the pin operates in high-impedance mode. Although Xilinx does not disclose the internal geometry (W/L ratios) of these MOSFETs, their macroscopic behavior is equivalent to a CMOS tri-state buffer with an effective on-resistance that is typically in the 20–40 Ω range for common I/O standards. This internal composition is significant because CMOS devices are well-known to exhibit measurable degradation under electrical and thermal stress, through mechanisms such as bias temperature instability (BTI), hot-carrier injection (HCI), time-dependent dielectric breakdown (TDDB), and interface-trap generation. These mechanisms alter carrier mobility and threshold voltage, which directly affects the small-signal and large-signal resistance of the I/O path. Therefore, any systematic drift in I/O impedance under prolonged thermal bias is naturally expected to originate from transistor-level degradation within this CMOS tri-state structure rather than from macroscopic packaging effects, unless clear evidence of solder intermittency or crack propagation is observed [3,4,7].

### 1.2. Physics of CMOS I/O Aging in FPGAs

CMOS I/O circuitry in FPGAs is subject to several electrical aging mechanisms that interact differently under thermal and bias stress. BTI is typically the most dominant in thick-oxide I/O transistors, causing charge trapping at the oxide–semiconductor interface and within the high-k dielectric. These traps modify the threshold voltage and transconductance, reducing drive strength over time. HCI, by contrast, arises when carriers gain sufficient kinetic energy under high V_ds_ to become injected into the gate oxide, producing permanent interface states. While BTI exhibits partial recovery when stress is removed, HCI is largely irreversible. Time-dependent dielectric breakdown (TDDB) is another mechanism, though it is less pronounced in I/O devices due to thicker oxide layers; it produces exponential increases in leakage current and eventual catastrophic failure, rather than gradual resistance drift.

In FPGAs, the combined influence of BTI and HCI manifests in measurable changes to I/O slew rates, timing margins, and static output resistance. Prior studies have shown that such degradation can accumulate across months or years of field operation, particularly in systems operating at elevated ambient temperatures or with sustained DC bias on unused I/Os. The SelectIO architecture in Spartan-6 employs complementary MOSFET drivers that directly modulate the impedance presented at the pad, meaning that threshold-voltage shifts or mobility degradation translate directly to measurable increases in the series resistance, R_path_. This connection between transistor-level physics and measurable macroscopic parameters forms the conceptual basis for interpreting the experimental data presented in this work.

The broader reliability research community has increasingly acknowledged that FPGA wear-out is no longer dominated solely by configuration SRAM soft errors or metallization failures; instead, transistor aging has become a central concern as nodes have scaled. Long-term stability of I/O circuits is particularly critical in applications requiring impedance matching, termination accuracy, or precise analog interfacing. The results presented here contribute to this emerging understanding by showing that measurable electrical drift occurs in older planar CMOS nodes, even without extreme voltage stress or overstress events, emphasizing that BTI must be accounted for in lifetime prediction models.

In this work, we investigate the degradation of I/O paths in a Xilinx Spartan-6 XC6SLX45-CSG324C FPGA subjected to two stress conditions: (1) thermal cycling between 80 and 140 °C and (2) constant high-temperature bias in the range of 80–120 °C. Analysis of the voltage-drop and resistance evolution reveals fundamentally different trends for the two modes. Thermal cycling exhibits a logarithmic degradation trajectory characteristic of package-level fatigue, whereas constant high-temperature bias produces a clear power-law dependence that is consistent with BTI-induced transistor aging. These results indicate that the dominant degradation mechanism is not solder joint or interconnect failure, but rather a BTI-driven Vth shift occurring within the I/O buffer transistors.

## 2. Theoretical Background

The long-term reliability of FPGA systems is strongly influenced by both package-level degradation and transistor-level electrical aging. In a typical FPGA I/O path, the electrical signal passes through solder joints, PCB pads, vias, board traces, and finally, the on-chip CMOS input buffer. Any degradation in these elements can lead to measurable changes in voltage drop or apparent line resistance, making electrical characterization a useful tool for identifying early failure mechanisms.

Thermal cycling is one of the dominant stress factors affecting package-level reliability. Numerous studies have shown that cyclic exposure to high and low temperatures produces thermo-mechanical strain, due to mismatches in thermal expansion between silicon, copper pads, and solder alloy, which can initiate or accelerate crack formation when sufficient strain accumulates [10]. In thermal-cycling environments, the rate of degradation correlates with strain-range effects, and many studies have shown that solder fatigue can be accurately described using Coffin–Manson-type power-law relationships, particularly for lead-free joints subjected to cyclic temperature excursions [11,12]. Similar observations were made in EBGA (Enhanced Ball Grid Array) packages, where combined temperature cycling and board flexure accelerate the initiation of cracks around the copper-metallized silicon interface, reducing the overall interconnect lifetime [13].

Previous studies have shown that FPGA packages are highly sensitive to transient opens and resistance spikes at their solder joints and I/O connections, especially under thermal and mechanical stresses. FPGA-based monitoring platforms have been used to detect these microsecond-scale intermittent faults, confirming that degradation mechanisms such as solder fatigue and interconnect intermittency can occur on very fast time scales. This highlights the importance of understanding the degradation behavior of FPGA I/O pins in reliability environments [14].

In addition to mechanical fatigue, semiconductor devices experience electrical aging when subjected to prolonged temperature and bias conditions. High-temperature operation can accelerate changes in threshold voltage, leakage current, and conduction behavior within MOS devices, often following continuous monotonic profiles such as logarithmic or power-law evolutions. Experimental analyses of MOSFET reliability commonly report bias-dependent shifts in threshold voltage, gate leakage, and oxide field distribution, especially when devices are stressed under high-temperature reverse bias or gate bias conditions [15]. These drifts reflect electronic processes such as charge trapping, interface-state activation, or oxide-field-induced degradation, which are fundamentally different from the abrupt mechanical cracking observed in solder joints.

Because thermal–mechanical fatigue and electrical aging arise from distinct physical processes, they also produce different electrical signatures. Thermal cycling typically causes gradual resistance changes due to micro-crack initiation and growth in the solder or interconnect layers, whereas constant temperature with sustained electrical bias tends to induce smoother, bias-dependent electronic drifts in semiconductor devices. Understanding the differences between these mechanisms is essential for correctly interpreting FPGA degradation behavior, particularly when analyzing resistance or voltage-drop changes in I/O paths under controlled stress conditions.

The following sections outline the structure of this study. Section 3 describes the experimental materials and methods used to implement the thermal-stress measurement setup, including instrumentation, data acquisition, and test procedures. Section 4 presents the results, along with a detailed discussion of the observed behavior under thermal loading. Finally, Section 5 provides the conclusions drawn from this work.

## 3. Materials and Methods

**Figure 2:**
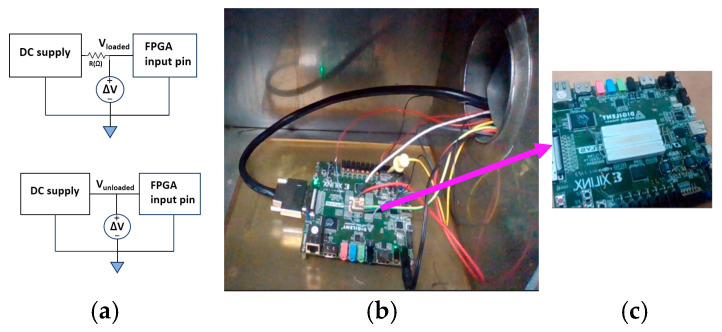
Experimental setup used for thermal-stress characterization, showing (**a**) the electrical schematic with a DC supply, series power resistor (250 Ω), and voltage measurement nodes for Vloaded and Vunloaded; (**b**) the FPGA board operating inside the thermal chamber during heating; and (**c**) a close-up view of the Digilent FPGA (Digilent, Pullman, WA, USA) board with the ceramic power resistors mounted on the device.

Further details about the testing orientation can be found in earlier studies [16,17]. Each pin was sequentially biased, using a DC supply (Rigol Technologies, Suzhou, China) connected in series with a high-power ceramic load resistor mounted inside the chamber. The resistor established a stable load, and its low temperature coefficient ensured that any change in voltage or current originated from the FPGA path rather than from external components. One terminal of the ceramic resistor was tied to the supply, while the other terminal was connected to the FPGA I/O pin under test, as shown in Figure 2. For every stressed pin, two measurements were taken: (i) the loaded voltage (V_load_), recorded when the resistor was connected to the pin and current flowed into the FPGA input buffer, and (ii) the unloaded voltage (V_unload_), collected when the resistor was disconnected, providing a baseline that reflects the natural I/O pin input characteristics without loading. A precision digital multimeter (DMM) was connected in series to measure the current simultaneously, allowing the effective resistance of the entire I/O path to be calculated from

(1) $$R_{\mathrm{path}}=\frac{V_{\mathrm{load}}}{I}$$

The durations of the two stress profiles were selected based on the characteristic time scales of the underlying degradation mechanisms, rather than on equal exposure durations. The constant high-temperature stress was applied for approximately 250 h to enable clear observation of the time-dependent resistance drift and to support reliable extraction of activation energy under steady-state bias conditions. In contrast, the thermal cycling experiment was limited to 300 cycles because thermomechanical degradation mechanisms, such as solder fatigue, typically manifest through intermittent resistance fluctuations or discontinuities once crack initiation begins.

In the present study, resistance traces during thermal cycling remained smooth and continuous throughout the full test duration, with no indication of intermittency or abrupt changes. This behavior indicates that extending the number of cycles would not have altered the dominant degradation mechanism under the applied stress conditions. Therefore, the selected durations were sufficient to differentiate between transistor-level aging and package-level thermomechanical effects while maintaining practical experimental timescales.

### 3.1. Electrical Configuration of the I/O Pins During Measurement

During all experiments, the FPGA I/O pins under investigation were configured as actively driven output buffers operating in a static DC mode. Each pin was programmed as a digital output with a fixed logic state, while internal pull-up and pull-down resistors were disabled to prevent unintended biasing effects. The output-enable signal remained asserted throughout the entire measurement process to ensure that the internal CMOS output transistors remained electrically active during both the loaded and unloaded measurement conditions.

For the loaded condition, the output pin sourced a current through the external high-power ceramic resistor connected to the supply rail, establishing a controlled current path through the I/O buffer and associated interconnects. For the unloaded condition, the external resistor was disconnected, while the internal electrical configuration of the I/O buffer remained unchanged. This ensured that the only difference between the two measurement states was the presence or absence of the external load.

The I/O standard, drive strength, and slew-rate configuration were fixed across all experiments to ensure repeatability. No dynamic signal toggling was applied; all measurements were performed under steady-state DC bias conditions. This configuration isolated degradation mechanisms associated with bias temperature stress and minimized any contribution from transient or switching-related effects.

Thermal stress was applied using a programmable thermal chamber arrangement. Two stress profiles were evaluated: (a) high-temperature soaking, where the FPGA was maintained continuously between 80 and 120 °C, and (b) thermal cycling, where the temperature was repeatedly swept between 80 and 140 °C. The board remained powered throughout testing, and V_load_, V_unload_, and the current were recorded automatically by the LabVIEW (version 2024 Q3 (64-bit) 24.3f0) system for the entire duration of the experiment at regular intervals to capture the resistance evolution as a function of time and temperature. All data were logged externally to avoid the thermal drift of measurement electronics. The long-term behavior of the extracted resistance traces was then analyzed to determine the dominant degradation mechanism.

**Figure 3:**
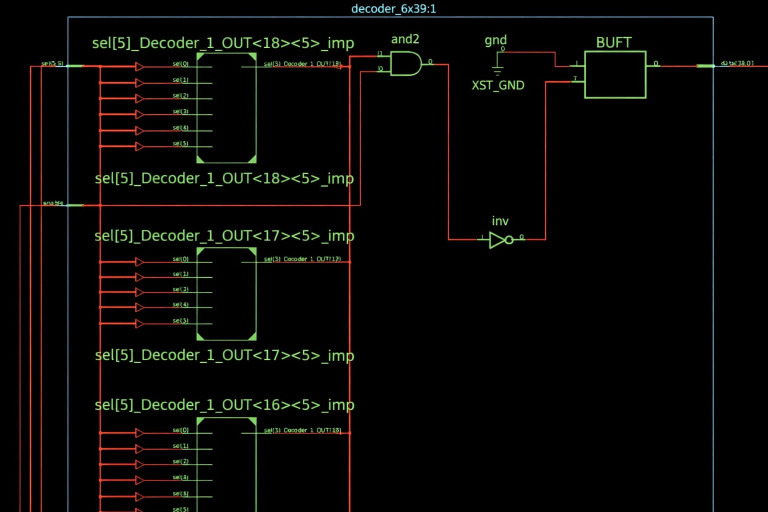
A partial view of the schematic of the sequencing development. The BUFT is positioned in the upper right corner.

The experiments were performed on a Digilent Nexys-3 (Digilent) development board incorporating a Xilinx Spartan-6 XC6SLX45-CSG324C FPGA (Xilinx), which provides three hundred and twenty-four I/O pins in a fine-pitch BGA package. To study degradation of the I/O interconnection paths—including solder joints, pads, package interconnects, and input-buffer transistors—thirty-nine pins were selected and routed out of the thermal chamber through insulated feedthroughs. The input buffer, type BUFT, is shown in a part of the FPGA development schematic from the Xilinx AMD ISE 14.7 software in Figure 3, below. The schematic shows the BUFT at the interface between the interconnects and the die. All I/O pins are connected to separate BUFT gates.

### 3.2. Electrical Interpretation of the Measured R_path_ and Its Connection to CMOS Aging

To understand the origin of the observed resistance drift, the measured quantity, $R_{\mathrm{path}}$, can be expanded into its constituent series elements:

(2) $$R_{\mathrm{path}}=R_{\mathrm{PCB}}+R_{\mathrm{via}}+R_{\mathrm{solder}}+R_{\mathrm{package}}+R_{\mathrm{pad}}+R_{\mathrm{ESD}}+R_{\mathrm{CMOS}}$$

Here, $R_{\mathrm{PCB}}$, $R_{\mathrm{via}}$, $R_{\mathrm{solder}}$, $R_{\mathrm{package}}$, and $R_{\mathrm{pad}}$ represent the static contributions of the board and package parasitics, all of which exhibit very small temperature coefficients (<0.1%/°C) and do not undergo monotonic drift under constant electrical bias. Since the thermal-stress profiles used in this study do not induce mechanical bending or cyclic strain, these parasitic components remain effectively time-invariant. Therefore, the only term with significant temperature- and bias-dependence is $R_{\mathrm{CMOS}}$: the effective on-resistance of the CMOS transistors inside the Spartan-6 tri-state I/O cell.

Quantitatively, the temperature coefficients of resistance (TCR) for typical PCB copper traces and solder interconnects are well-established in the literature and are generally on the order of 0.003–0.004 °C^−1^ for copper and below 0.001 °C^−1^ for common lead-free solder alloys. Over the temperature range explored in this study (up to approximately 150 °C), these coefficients correspond to reversible resistance variations of only a few percent and do not produce cumulative or time-dependent drift once thermal equilibrium is reached.

Moreover, degradation mechanisms such as solder fatigue or interconnect cracking require cyclic mechanical strain, which is typically induced by board-level flexure, vibration, or large differential thermal expansion. Under the experimental conditions used here, the device was mounted on a mechanically stable platform and subjected only to uniform thermal exposure, without applied mechanical loading. As a result, the conditions necessary to initiate solder fatigue or interconnect damage were not present.

The absence of transient resistance spikes, discontinuities, or non-monotonic behavior in the measured data further supports the conclusion that PCB traces and solder joints remain electrically stable throughout the experiment. Consequently, the observed resistance evolution can be attributed primarily to intrinsic device-level effects—most notably, transistor aging mechanisms such as bias temperature instability—rather than to time-dependent degradation of external interconnects.

Under BTI, trapped charge at the gate dielectric increases the transistor threshold voltage and reduces carrier mobility (μ), producing a measurable increase in the conduction resistance:

(3) $$R_{\mathrm{CMOS}}\propto \frac{1}{\mu (V_{GS}-V_{th})}$$

A BTI-induced shift of ≈30–60 mV or a modest mobility reduction (5%) is sufficient to increase the effective on-resistance of a 20–40 Ω I/O driver by approximately 0.5–1 Ω, which is fully consistent with the resistance evolution measured during the high-temperature stress intervals. Because package-level elements do not show such monotonic drift, the agreement between the predicted and measured values strongly supports a transistor-level degradation mechanism, rather than a solder-fatigue or packaging-failure mode.

## 4. Results and Discussion

The Spartan-6 FPGA is fabricated in a 45 nm CMOS process, and all internal logic blocks—including LUTs, routing switches, configuration SRAM, and I/O buffers—are constructed from complementary MOSFET (CMOS) devices. Each I/O pin contains a CMOS input buffer and a tri-stateable CMOS output driver. The tri-state stage allows the pin to operate as input, output, or high impedance, depending on its configuration. Because these structures rely on n- and p-channel MOSFETs, the I/O path is directly affected by transistor-level degradation mechanisms such as bias-temperature instability (BTI), hot-carrier injection (HCI), and interface-state generation. This transistor-based composition explains why the observed resistance drift during thermal stress follows BTI-like behavior, rather than solder joint intermittency, which would originate from package-level mechanical effects rather than CMOS device aging.

**Figure 4:**
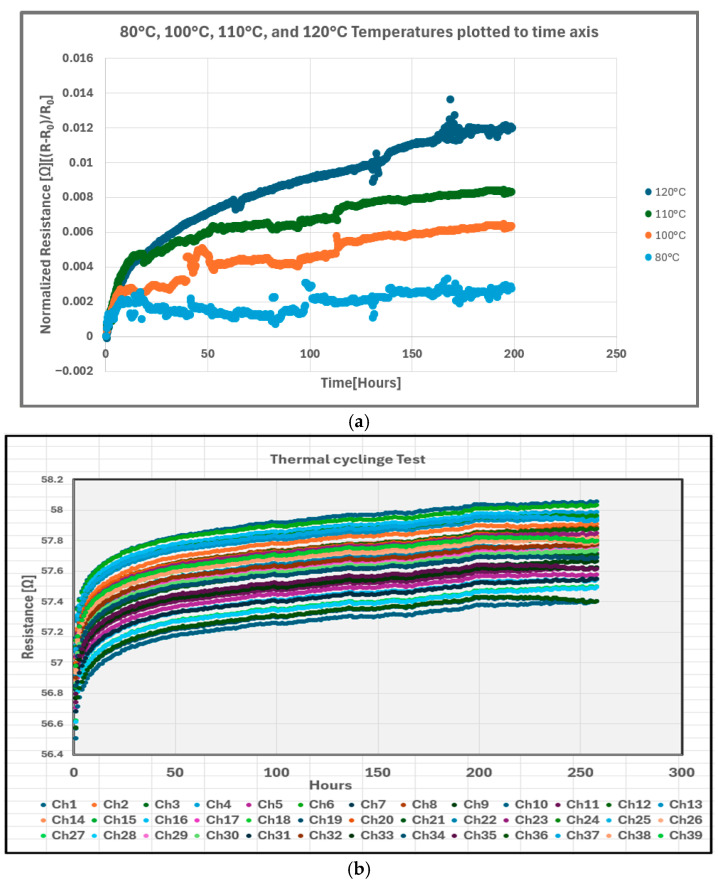
Resistance evolution of the Spartan-6 FPGA I/O paths under thermal stress. (**a**) High-temperature static bias degradation measured across single pin at 80–120 °C, showing smooth, monotonic resistance increase consistent with BTI-driven transistor aging and absence of solder joint intermittency. (**b**) Thermal-cycling degradation (ΔT up to 150 °C) across 39 channels, exhibiting logarithmic resistance growth with cycle count, indicating no solder-fatigue initiation under cyclic thermal loading.

The degradation behavior of the Spartan-6 FPGA I/O paths was evaluated under high-temperature static bias (80–120 °C) and thermal cycling (ΔT up to 150 °C), using resistance monitoring, Weibull modeling, and Arrhenius activation-energy extraction. The resistance-versus-time measurements under static stress, shown in Figure 4a, exhibit a smooth monotonic increase across all 39 monitored pins with no intermittent spikes or abrupt jumps, indicating the absence of solder joint cracking or metallurgical intermittency. Instead, the resistance follows a power-law time dependence, which is consistent with bias temperature instability (BTI) and interface-state generation within the MOS input-buffer transistors. Similar BTI-driven Vth drift has been widely documented in CMOS and FPGA technologies, including 40 nm Xilinx platforms examined by Sim et al. [18], long-term FPGA aging analyses showing BTI/HCI dominance, and interface-trap formation mechanisms described in MOS reliability studies [3,4,6,15]. In contrast, the thermal-cycling resistance curves shown in Figure 4b display a logarithmic degradation trend, yet remain smooth and continuous over 300 cycles, with no transient opens typically associated with solder joint fatigue. Prior solder-intermittency studies using SJ-BIST demonstrated that cracked joints produce microsecond-scale resistance spikes [14], none of which were observed here. Furthermore, BGA package-reliability studies confirm that thermal cycling without mechanical bending or flexure rarely induces solder joint cracking [10,13], which is consistent with the stable behavior observed in this work.

### 4.1. BTI-Driven Resistance Drift in CMOS I/O Structures

BTI is a dominant reliability mechanism in CMOS technologies, arising from the trapping of carriers in the gate dielectric and at the Si–SiO_2_ interface. Under negative gate bias, PMOS devices experience NBTI, whereas NMOS devices experience PBTI under positive gate bias. Both effects cause a Vth shift that is proportional to stress time, following either a power-law or reaction–diffusion model. The resulting reduction in the drive current leads to an increase in the effective on-resistance of the tri-state buffer MOSFETs. BTI is strongly temperature-accelerated, with the reported activation energies ranging from 0.1 to 0.5 eV in 45–65 nm CMOS. The E_a_ ≈ 0.62 eV extracted in this work falls within this range, supporting the suggested conclusion that BTI dominates the observed degradation under static high-temperature stress. Furthermore, the smooth, monotonic evolution of resistance and the lack of transient events are a known characteristic of BTI aging, rather than abrupt failure mechanisms. No transient resistance spikes or abrupt discontinuities were observed in any of the measured channels during thermal cycling. The voltage and current were continuously recorded using a LabVIEW-based data acquisition system, providing sufficient temporal resolution to capture the resistance fluctuations typically associated with solder joint intermittency. Prior studies have shown that solder fatigue produces resistance excursions on time scales ranging from milliseconds to seconds once crack initiation begins. Such behavior would therefore be observable within the temporal resolution of the present measurements.

Moreover, the resistance traces remained smooth and continuous throughout the experiment, without stochastic fluctuations or intermittent behavior. This indicates that no crack initiation or intermittent contact occurred during testing. While extremely short-lived microsecond-scale events cannot be completely excluded, such events are typically associated with advanced crack propagation and would be accompanied by pronounced instability in resistance trends, which was not observed in this study.

Similar resistance drift patterns and BTI behavior have been reported in FinFET FPGAs and older planar CMOS nodes, demonstrating that the results observed in Spartan-6 are fully consistent with the established CMOS reliability theory [6,18].

**Figure 5:**
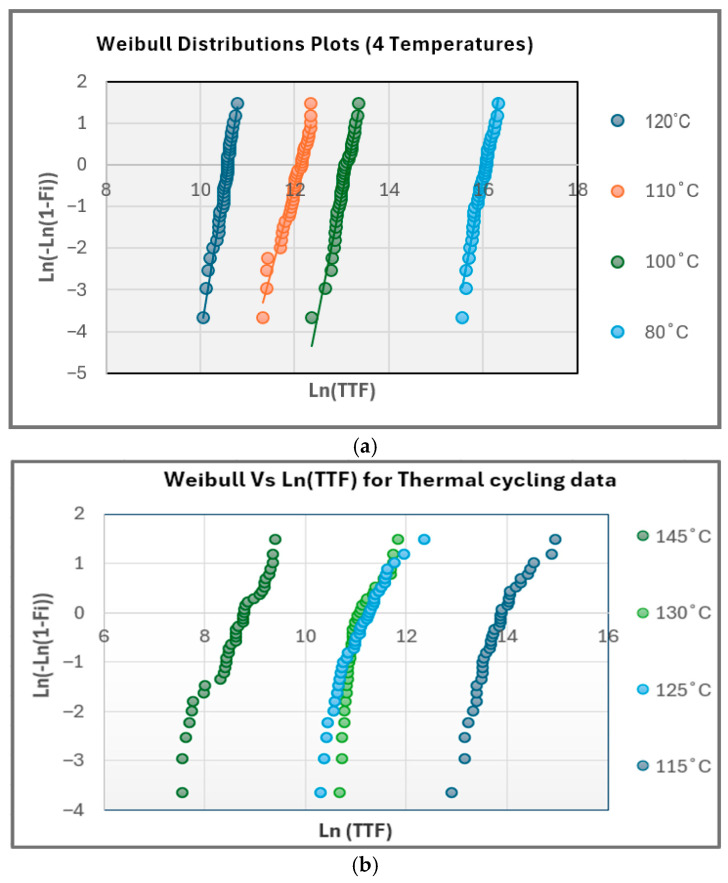
Weibull time-to-failure (TTF) distributions for FPGA I/O degradation under thermal stress. (**a**) Static high-temperature Weibull plots (80–120 °C), showing linear behavior, parallel slopes, and temperature-dependent leftward shifts that are characteristic of a single BTI-dominated degradation mechanism. (**b**) Thermal-cycling Weibull plots exhibiting slightly lower β slopes and broader scatter.

The Weibull time-to-failure distributions shown in Figure 5 further clarify the mechanism. For static stress (80–120 °C), as shown in Figure 5a, the Weibull plots exhibit excellent linearity and nearly parallel slopes, shifting left with increasing temperature, which indicates strong thermal acceleration of a single degradation mechanism. The linearity and parallelism of the Weibull plots indicate a single dominant degradation mechanism, which is consistent with MTOL-based reliability interpretations where distinct failure modes manifest as slope or curvature deviations [1]. The increase in Weibull slope (β) at higher temperatures reflects reduced scatter and a uniform BTI-dominated failure mode, which is consistent with the findings of Sim et al. [18] and FPGA aging studies. Conversely, the Weibull distributions for thermal cycling (Figure 5b) display broader scatter and slightly lower β values, reflecting a mild thermomechanical influence but still retaining linear behavior without curvature or β < 1. Such curvature and early-life scatter are characteristic of Coffin–Manson solder-fatigue behavior under strain-controlled thermal cycling [11,12]; thus, their absence confirms that no fatigue-driven solder cracking occurred. The classical temperature-based Coffin–Manson model is expressed as follows:

(4) Nf = A(ΔT)^−n^

where N_f_ is the number of cycles to failure, A is the material/geometry constant, ΔT is the temperature swing per cycle and n is the fatigue exponent (temperature acceleration factor). The Coffin–Manson analysis presented in this section is included to quantitatively evaluate and exclude it as a primary contributor, not to suggest that solder fatigue is the dominant degradation mechanism in the present study. Thermal cycling is a well-established driver of solder joint fatigue and therefore, its potential influence must be assessed when interpreting resistance evolution under cyclic thermal stress. By applying the Coffin–Manson framework, this work establishes whether the observed degradation behavior is consistent with classical thermomechanical fatigue or whether alternative mechanisms dominate.

As shown in the following analysis, the extracted parameters and trends do not align with those expected for solder-fatigue-driven failure. Instead, the results demonstrate that the measured behavior deviates significantly from classical Coffin–Manson predictions, supporting the conclusion that transistor-level aging mechanisms, rather than interconnect fatigue, govern the observed degradation. Thus, the Coffin–Manson analysis serves as a diagnostic tool to rule out solder joint fatigue, rather than as a model describing the dominant failure process.

**Figure 6:**
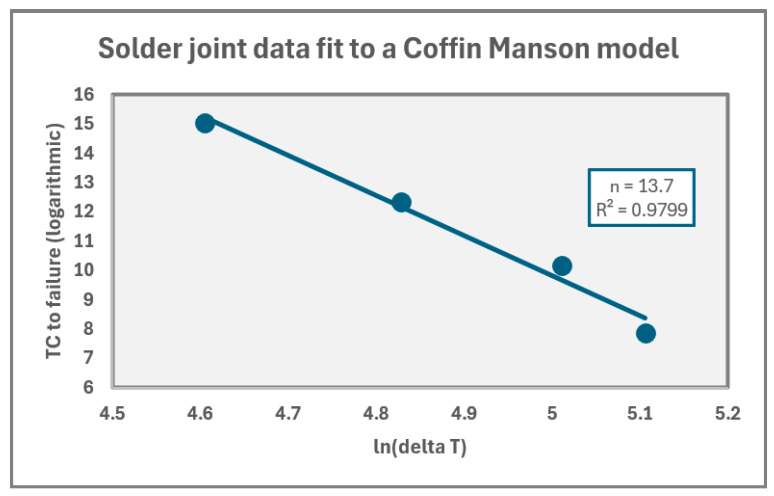
“n” coefficient extraction for thermal cycling data.

Our measured data do not exhibit early-life failures, nonlinear Weibull curvature, or sensitivity to ΔT that are consistent with this model, solder-fatigue-driven cracking is unlikely. Figure 6 displays the thermal cycling data applied to the Coffin–Manson model. The results show an “n” coefficient of 13.7. The value is far beyond the expected “n” coefficients published in JEDEC standards [19]. This implies that the degradation observed in the study cannot be characterized as dominantly solder joint fatigue.

**Figure 7:**
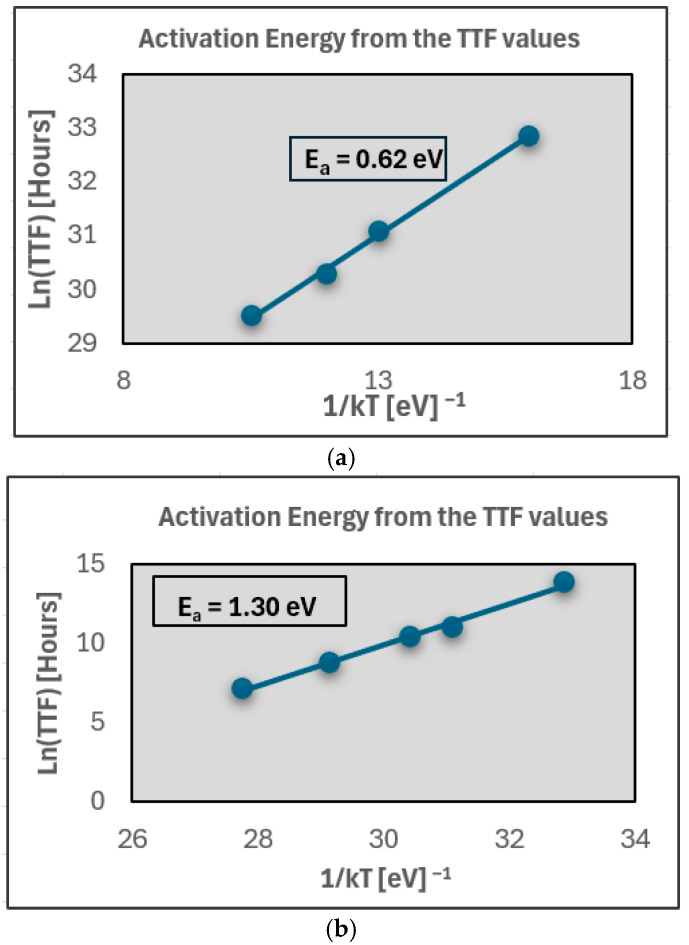
Arrhenius activation-energy extraction for FPGA I/O degradation. (**a**) Static high-temperature bias condition yielding an activation energy of E_a_ ≈ 0.62 eV, consistent with BTI-induced charge trapping and interface-state generation in CMOS devices. (**b**) Thermal-cycling condition yielding a higher apparent activation energy of E_a_ ≈ 1.30 eV, which is characteristic of thermomechanical strain and creep effects, but without corresponding solder-fatigue signatures in resistance or Weibull plots.

The activation-energy analysis shown in Figure 7 further distinguishes the underlying mechanisms. Static high-temperature stress yields an activation energy of E_a_ ≈ 0.62 eV, which lies within the widely reported BTI activation-energy range (0.1–0.5 eV) for MOSFETs and FPGA devices [3,4,6,15,16,18], strongly supporting BTI-induced transistor aging as the dominant degradation mechanism under constant bias [20]. While the extracted activation energy of approximately 0.62 eV is the most consistent with bias temperature instability, contributions from other degradation mechanisms—particularly hot-carrier injection (HCI)—cannot be entirely excluded. HCI is known to induce interface-state generation and mobility degradation under conditions of elevated electric field and carrier energy, and it can contribute to long-term reliability degradation in CMOS devices. However, HCI effects are typically more pronounced under dynamic switching conditions and high drain-to-source voltages, rather than under the quasi-static bias conditions applied in this study.

In the present experiments, the I/O buffers were operated under steady DC bias without high-frequency signal transitions, significantly limiting the likelihood of strong HCI contributions. Furthermore, reported activation energies associated with HCI-related degradation often differ from those typically observed for BTI-dominated processes. The smooth, monotonic resistance evolution and the extracted activation energy therefore suggest that BTI is the dominant degradation mechanism, while any contribution from HCI is expected to be secondary. This interpretation is consistent with prior CMOS reliability studies reported in the literature.

The thermal-cycling data produce a higher activation energy of E_a_ ≈ 1.30 eV, which is consistent with thermomechanical strain accumulation, creep, and relaxation processes in packaging materials [10,11,12,13]. However, because resistance traces remain smooth, Weibull plots remain linear, and no intermittency is detected. This higher E_a_ is interpreted as secondary thermomechanical modulation, rather than solder-fatigue-driven damage. Overall, the resistance evolution (Figure 4a,b), Weibull behavior (Figure 5a,b), and Arrhenius activation-energy results (Figure 7a,b) collectively indicate that BTI-driven transistor-level aging—not solder joint or package degradation—is the dominant failure mechanism in Spartan-6 I/O paths under both static and cyclic thermal stress conditions, with no electrical signatures that are consistent with solder joint cracking observed.

### 4.2. Absence of Solder-Joint Fatigue and Role of Board-Level Flexure

Thermal cycling commonly induces solder joint fatigue when accompanied by significant board-level flexure or when the package experiences differential thermal expansion that is sufficient to generate shear strain at the solder interface. However, in the current study, the FPGA was mounted on a mechanically rigid PCB and subjected to temperature cycling without bending forces or vibration. The literature on BGA reliability shows that crack initiation generally requires either high ΔT, mechanical shock, or flexural displacement of the PCB [11,13]. In the absence of these conditions, solder joints typically remain intact, even across hundreds of cycles.

The resistance data in Figure 4a,b show no microsecond-scale resistance spikes, which are the hallmark of intermittent opens caused by crack breathing in fatigued solder balls, as documented by Hofmeister et al. [14]. Likewise, the Weibull distribution exhibits no β < 1 region or curvature, both of which are signatures of Coffin–Manson fatigue initiation. Instead, the cycling results display shallow yet linear Weibull slopes and smooth resistance trends, indicating mild thermomechanical effects but no crack formation. These observations strongly support the conclusion that the measured degradation arises from on-die CMOS mechanisms, rather than package-level fatigue.

### 4.3. Detailed Reliability Mechanism Comparison Between CMOS Aging and Package-Level Degradation

To place the observed degradation behavior in a broader context, this section compares the present findings with existing studies on CMOS aging and package-level reliability mechanisms. A key challenge in interpreting degradation signatures in FPGA I/O paths is distinguishing between on-die transistor aging and external metallurgical or solder joint degradation mechanisms. Although the total measured resistance includes contributions from the PCB trace, BGA solder joints, package substrate, and on-die CMOS circuitry, only a subset of these elements exhibits bias- and temperature-dependent aging behavior. Solder joints, for example, typically degrade through crack initiation and propagation driven by cyclic shear strain, leading to intermittent opens, transient resistance spikes, and nonlinear acceleration of degradation with increasing thermal excursion [21,22,23]. Such behavior is commonly associated with curvature in Weibull distributions and early-life failure characteristics [14,21,22].

In the present study, none of these signatures were observed. The resistance evolution under both static and cyclic thermal stress remained smooth and continuous, with no intermittent discontinuities or evidence of crack-driven behavior. The absence of β < 1 regimes or early-life scattering in the Weibull distributions further indicates that solder joint fatigue was not activated under the applied stress conditions. These observations are consistent with prior studies showing that solder fatigue typically requires large temperature excursions (often exceeding 200 °C) and/or significant mechanical flexure to initiate crack formation [21,22,23]. Under the present experimental conditions, where thermal cycling was applied without board-level bending, solder joints are therefore expected to only experience elastic deformation, rather than progressive mechanical damage.

In contrast, degradation mechanisms associated with CMOS transistors—such as bias temperature instability (BTI) and hot-carrier injection (HCI)—produce smooth, time-dependent resistance evolution governed by charge trapping and interface-state generation [24,25,26,27,28,29]. These mechanisms are strongly temperature- and bias-dependent but do not generate abrupt resistance discontinuities. Because the Spartan-6 SelectIO driver consists of complementary MOSFETs with effective on-resistances on the order of tens of ohms, even modest mobility degradation (on the order of a few percent) leads to measurable changes in the overall I/O path resistance. The observed activation energy of approximately 0.62 eV under static bias is fully consistent with the reported BTI values for thick-oxide CMOS technologies in the 40–65 nm node range, further supporting transistor-level aging as the dominant degradation mechanism.

Thermal cycling produced higher apparent activation energy (~1.3 eV), which is consistent with thermomechanical strain relaxation and creep processes, rather than solder joint cracking. Importantly, this elevated activation energy was not accompanied by intermittent resistance behavior or Weibull curvature, indicating that thermomechanical effects act as a secondary modulation, rather than a primary failure mechanism. This distinction is critical, as it demonstrates that thermal cycling alone—when applied without mechanical loading—does not induce solder joint fatigue in the tested FPGA package.

Compared with prior studies on FPGA and power-device reliability, which often focus exclusively on either transistor-level aging or package-level failure, the present work provides a unified experimental framework that is capable of distinguishing between these mechanisms. By combining resistance-based monitoring with Weibull analysis and activation-energy extraction, this study enables clear separation of electrical aging from mechanical degradation processes. This integrated approach extends existing reliability models and improves the predictive capability for the long-term behavior of FPGA I/O structures under realistic operating conditions.

## 5. Conclusions

This work presented a comprehensive reliability investigation of FPGA I/O paths subjected to constant high-temperature stress and thermal cycling. Using resistance-based monitoring, Weibull statistical analysis, and activation-energy extraction, the dominant degradation mechanisms governing long-term behavior were systematically identified.

Under static high-temperature stress, the resistance evolution exhibited smooth, monotonic behavior that was consistent with bias temperature instability (BTI). The extracted activation energy of approximately 0.62 eV aligns well with the reported values for BTI in CMOS technologies, confirming transistor-level aging as the dominant degradation mechanism. Under thermal cycling, resistance traces remained continuous and free of intermittent behavior, indicating the absence of solder joint fatigue or interconnect cracking. Although a higher apparent activation energy was observed under cyclic stress, this behavior is attributed to secondary thermomechanical effects, rather than crack-driven failure.

Overall, the results demonstrate that BTI-driven transistor aging dominates degradation in FPGA I/O structures under both static and cyclic thermal stress, while package-level degradation remains secondary under the investigated conditions. These findings are consistent with the existing reliability literature and provide valuable insight into the long-term behavior of commercial off-the-shelf FPGA devices operating in thermally demanding environments.

Future work will extend this study to include combined electrical overstress, mechanical loading, and environmental factors, as well as advanced characterization techniques such as in situ monitoring and targeted microstructural analysis. Such efforts will further enhance the understanding of degradation mechanisms and support the development of more accurate lifetime prediction models for advanced electronic systems.

## Data Availability

The original contributions presented in this study are included in the article. Further inquiries can be directed to the corresponding author.

## References

[1] Bernstein J.B., Bensoussan A., Bender E. (2017). Reliability prediction with MTOL. Microelectron. Reliab..

[2] Bender E., Bernstein J.B., Boning D.S. (2024). Modern Trends in Microelectronics Packaging Reliability Testing. Micromachines.

[3] Goetzberger A., Lopez A.D., Strain R.J. (1973). On the Formation of Surface States during Stress Aging of ThermalSi-SiO_2_ Interfaces. J. Electrochem. Soc..

[4] Fair R., Sun R. (1981). Threshold-voltage instability in MOSFET’s due to channel hot-hole emission. IEEE Trans. Electron Devices.

[5] Lloyd J.R. (2016). The Lucky Electron Model for TDDB in Low-k Dielectrics. IEEE Trans. Device Mater. Reliab..

[6] Sobas J., Marc F. (2024). Degradation Measurement and Modelling under Ageing in a 16 nm FinFET FPGA. Micromachines.

[7] Smerdon M. (2011). High-Volume Spartan-6 FPGAs: Performance and Power Leadership by Design (WP396).

[8] Xilinx (2011). Spartan-6 Family Overview (DS160); Rev. 2.0.

[9] Keller A.M., Wirthlin M.J. (2021). Partial TMR for Improving the Soft-Error Reliability of SRAM-Based FPGA Designs. IEEE Trans Nucl. Sci..

[10] Mattila T.T., Simecek J., Kivilahti J.K. Failure Modes of Solder Interconnections under Mechanical Shock Loading at Elevated Temperatures. Proceedings of the 1st Electronic Systemintegration Technology Conference.

[11] Engelmaier W. (1983). Fatigue Life of Leadless Chip Carrier Solder Joints during Power Cycling. IEEE Trans. Components, Hybrids, Manuf. Technol..

[12] Yu Q., Shiratori M. (2005). Thermal Fatigue Assessment of Lead-Free Solder Joints. https://hal.science/hal-00189474v1.

[13] Yang L., Bernstein J. (2008). Reliability Study of High-Density EBGA Packages Using the Cu Metallized Silicon. IEEE Trans. Compon. Packag. Technol..

[14] Hofmeister J.P., Lall P., Panchagade D., Roth N.N., Tracy T.A., Judkins J.B. Ball Grid Array (BGA) Solder Joint Intermittency Detection: SJ BIST. Proceedings of the IEEE Aerospace Conference.

[15] Anoldo L., Zanetti E., Coco W., Russo A., Fiorenza P., Roccaforte F. (2024). 4H-SiC MOSFET Threshold Voltage Instability Evaluated via Pulsed High-Temperature Reverse Bias and Negative Gate Bias Stresses. Materials.

[16] Bender E., Avraham T., Bernstein J.B. Statistical degradation in BGAs for early fault detection. Proceedings of the ISTFA 2024-50th International Symposium for Testing and Failure Analysis Conference.

[17] Bender E., Sitbon M., Avraham T., Gerasimov M. (2025). Spatial Monitoring of I/O Interconnection Nets in Flip-Chip Packages. Electronics.

[18] Li Z., Huang Z., Wang Q., Wang J., Luo N. (2022). Implementation of Aging Mechanism Analysis and Prediction for XILINX 7-Series FPGAs with a 28-nm Process. Sensors.

[19] JEDEC Solid State Technology Association (2017). Temperature Cycling.

[20] Dhyani M., Singh S., Tzhayek N., Bernstein J.B. (2025). Power-Law Time Exponent n and Time-to-Failure in 4H-SiC MOSFETs: Beyond Fixed Reaction–Diffusion Theory. Micromachines.

[21] Qiu B., Zhang M., Xu L., Zhou Y., Liu Y. (2022). Survey on Fatigue Life Prediction of BGA Solder Joints. Electronics.

[22] Lau J.H. (2021). State of the Art of Lead-Free Solder Joint Reliability. J. Electron. Packag..

[23] Depiver J.A., Mallik S., Amalu E.H. (2020). Effective solder for improved thermo-mechanical reliability of solder joints in a ball grid array (BGA) soldered on printed circuit board (PCB). J. Electron. Mater..

[24] Mahapatra S., Alam M.A., Kumar P.B., Dalei T.R., Varghese D., Saha D. (2005). Negative Bias Temperature Instability in CMOS Devices. Microelectron. Eng..

[25] Ťapajna M., Kuzmík J. (2020). Current. Understanding of Bias-Temperature Instabities in GaN and Si MIS-Devices. Crystals.

[26] Kaczer B., Grasser T. Ubiquitous Relaxation in BTI Stressing—New Evaluation and Insights. Proceedings of the 2008 IEEE International Reliability Physics Symposium.

[27] Stott E.A., Wong J.S.J., Sedcole P., Cheung P.Y.K. (2010). Degradation in FPGAs: Measurement and Modelling. Proceedings of the FPGA ‘10: ACM/SIGDA International Symposium on Field Programmable Gate Arrays.

[28] Yabuuchi M., Kobayashi K. (2012). NBTI-Induced Delay Degradation Analysis of FPGA Routing Structures. IPSJ Trans. Syst. LSI Des. Methodol..

[29] Naouss M., Marc F. Modeling Delay Degradation Due to NBTI in FPGA Look-Up Tables. Proceedings of the 26th International Conference on Field Programmable Logic and Applications (FPL).

